# Maternal distress during the COVID-19 outbreak: A socio-ecological perspective

**DOI:** 10.1371/journal.pone.0302266

**Published:** 2024-05-03

**Authors:** Mor Keleynikov, Noga Cohen, Joy Benatov

**Affiliations:** 1 Department of Special Education, Faculty of Education, University of Haifa, Haifa, Israel; 2 Edmond J. Safra Brain Research Center for the Study of Learning Disabilities, University of Haifa, Haifa, Israel; Kwame Nkrumah University of Science and Technology, GHANA

## Abstract

**Introduction:**

Mothers faced an increased risk of adverse mental health outcomes during the COVID-19 pandemic compared to other populations. However, there is little data on the factors that placed mothers at increased risk of distress.

**Aims:**

The present study explored a range of individual, familial, and environmental factors associated with psychological distress in mothers during the COVID-19 pandemic.

**Method:**

This repeated cross-sectional study was composed of a convenience sample of mothers who completed an online survey that included a demographic questionnaire, an emotion regulation questionnaire, and the Depression, Anxiety, and Stress scale. The survey was administered during the second and third lockdowns in Israel in 2020–2021.

**Results:**

The study included 575 mothers (M age = 39). The findings of a hierarchical regression indicated that individual-level factors, composed of age and emotion regulation tendencies predicted psychological distress. The family-level factors of household income and number of children in the family also predicted distress. In terms of environmental-level factors, COVID-19-related media consumption and school status (open or closed) were also significant predictors of psychological distress. Importantly, the results showed that the most important predictors of psychological distress in mothers during the COVID-19 outbreak were school closures, household income, and the use of adaptive and maladaptive emotion regulation strategies.

**Conclusions:**

The findings highlight the intersection of individual, familial, and environmental factors in mothers’ mental health during crises.

## Introduction

At the beginning of 2020, the COVID-19 virus spread rapidly across the globe, and was declared a global pandemic by the World Health Organization in March [1]. To prevent the pandemic from spreading, governments implemented a variety of steps that included the closing of non-essential services, travel restrictions, quarantines, and school lockdowns [2]. Although these restrictions were crucial to mitigating the spread of the disease, they also constituted a burden on the population. The pandemic affected parents in particular, who needed to take on a much more intense educational role while trying at the same time to deal with daily chores and work [2, 3]. Findings collected during the pandemic indicated that mothers tended to report higher stress levels and lower subjective well-being [3, 4], as well as higher levels of burnout and depressive symptoms than non-parents and fathers [5, 6]. Maternal psychological distress can impair job functioning, impede family interactions, affect children’s mental health, and may contribute to divorce and child mistreatment [6–8]. Since women are mostly responsible for childcare, with studies showing that mothers devote almost twice as much time to household and childcare responsibilities as fathers [9], the current study focused on mothers.

While studies have investigated the risk factors associated with mental distress in mothers during the COVID-19 pandemic, the majority have either concentrated primarily on pregnant women or failed to adopt a comprehensive, holistic approach to examining these factors [10]. Nevertheless, delving into the experiences of mothers is essential since the insights gleaned are likely to extend beyond the immediate context of the pandemic, and are applicable to other crises, including war and natural disasters. This broader understanding is instrumental not only to bolstering the resilience of individual families but also in terms of contributing to overall economic and communal stability. There is growing acknowledgment of the need to consider multi-level factors as contributors to mental health [11]. The current study thus drew on Bronfenbrenner’s Socio-Ecological theory [12] to identify the factors that contribute to mental health problems in mothers. This type of holistic approach not only explores individual coping resources but also encompasses broader contexts by examining factors at the family and environmental levels. In times of adversity, this type of investigation is needed to carry out and plan for more comprehensive mental health policies and interventions for mothers.

Socio-Ecological Theory posits that mental health is affected by the microsystem, mesosystem, exosystem, macrosystem, and chronosystem. The microsystem refers to individual-level factors, the exosystem covers family-level factors, the mesosystem includes factors within one’s proximal environment, and the macrosystem extends to wider social factors. Although the Socio-Ecological model has often been used to predict children’s mental conditions, it can also be applied to parents [13]. Previous studies have found that parents’ mental state is affected by multiple factors such as available mental resources, financial status, the quality of the marital relationship, and their offspring’s mental and physical health [13–15]. Therefore, investigating risk and resilience factors in mothers requires consideration of both individual and contextual factors, making Socio-Ecological Theory a suitable framework.

The current study thus implemented a social-ecological theoretical perspective to examine how factors at multiple levels of the socio-ecological system may account for the deleterious mental health effects of the COVID-19 outbreak on mothers. Below we briefly summarize the key factors identified in previous studies conducted during the COVID-19 pandemic that may be related to maternal stress. These include individual-level factors (e.g., age and emotion regulation tendencies), family‐level factors (e.g., relationship status, number of children in the family, household income, children’s age, and parenting a disabled child), and environmental-level factors (e.g., school status, media exposure, as well as the level of direct exposure to COVID-19).

### Individual-level factors

Studies on individual differences in the use of emotion regulation strategies have led to a better understanding of emotional distress in general, and during the COVID-19 pandemic in particular [16–18]. *Emotion regulation (ER)* refers to any process that influences the manifestation, intensity, or duration of an emotional response [19, 20]. ER tendencies can serve as significant predictors of parental outcomes during a pandemic, since they have been shown to moderate the association between specific personality traits [such as neuroticism] and mental health [18–20]. The two most common strategies individuals use to regulate their emotions are known as suppression and reappraisal [17]. Suppression entails inhibiting the behavioral expressions of emotions. Reappraisal is a cognitive strategy that entails reinterpreting a situation that evokes negative emotions more positively. While reappraisal is commonly considered to be an adaptive strategy related to resilience, suppression is usually viewed a non-adaptive strategy associated with psychological distress [17, 21]. In the context of the COVID-19 outbreak, mothers with a higher tendency to use reappraisal may have been able to see the situation in a more flexible and positive light, whereas the habitual use of suppression may have exacerbated the negative emotions experienced in situations resulting from the pandemic. Several studies conducted during peak COVID-19 periods found that parental use of adaptive ER strategies moderated the association between COVID-19-related stress and parental burnout [22], as well as children’s stress reactions [23]. A study on parents indicated that a reappraisal intervention could reduce parents’ stress levels during the COVID-19 outbreak [24].

In addition to ER, age is also considered to be an important predictor of maternal distress [25]. Specifically, older parents are considered to have better strategies to cope with distress [26]. During the COVID-19 pandemic, younger mothers were found to be at higher risk for mental distress symptoms than older mothers [27].

### Family-level factors

Within Socio-Ecological Theory, family‐level factors including pre-existing and pandemic-specific risk and resilience factors are considered to shape mothers’ mental state. For example, a study conducted during the COVID-19 outbreak found that children’s mental health and child-parent conflicts contributed to parenting stress [28]. Family-level factors may affect mothers’ well-being when they are associated with high demands (e.g., having young children, having many children), low resources (e.g., when the household income is low), or both (e.g., being a single mother, parenting a child with a disability). These family-level factors were found to increase vulnerability to parental distress during COVID-19 [29–31]. One of the main family-level factors considered to influence mothers’ mental state, especially during the COVID-19 pandemic, was relationship status [31–33]. Specifically, some of the most challenging aspects of the pandemic [e.g., childcare, financial concerns, restrictions on social life, and loneliness] were amplified for single parents who had to combine work and childcare without the support of a partner. A recent study showed that single parents tended to report more parental stress than partnered parents during the COVID-19 outbreak [4, 33]. In addition, being married mediated the association between children’s psychological symptoms and parental mental health [32]. Household financial strain is also a major source of stress for parents because of the unstable or unsafe situations it creates [27, 31]. During the pandemic, low-income families reported struggling to meet basic needs and arranging for childcare. They also reported suffering from other forms of money-related stress, such as worries about their financial status in the future [34].

The number of children in the family also plays a role in mothers’ mental health. For example, a study conducted during the COVID-19 outbreak on a large sample of more than 50,000 participants from 26 countries and territories found that increases in individuals’ perceived stress levels were directly correlated with the number of children in their households [4]. This may be because the financial and emotional burden is higher in families with many children. Children’s ages can also impact mothers’ mental state, such that parents of younger children tend to report greater difficulties than parents of older children [28, 35]. The developmental needs of younger children may have been more stressful and demanding for mothers during the pandemic because these children were entirely dependent on their caregivers during lockdowns and required constant supervision and parental involvement [36].

Finally, although the negative consequences of the pandemic were evident in mothers around the world [2, 6, 28], some familial constellations emerged as more vulnerable than others. Mothers of children with developmental disorders (DD) are one example, given their children’s difficulty coping with change and the discontinuation of treatments which were indispensable for these children [37]. The effects of COVID-19 on parents of children with DD have been examined in quite a few studies (for a review see [29]), in which parents of children with DD reported significantly greater anxiety, depression, stress, and a greater decrease in quality of life than parents of typically developing (TD) children [29, 30]. Furthermore, during the pandemic, parents of children with DD reported having difficulties dealing with their children’s behavior problems and feeling as though they could not meet their children’s needs at home [38]. They reported having concerns over the functional, social, and behavioral implications of the lockdowns on their children [29, 38].

### Environmental-level factors

Socio-Ecological Theory takes factors within an individual’s societal environment into account that can have both a direct and indirect impact on psychological distress [12]. Exposure to COVID-19 was an important environmental-level factor in terms of pandemic-related mental distress. According to Bridgeland and colleagues [39], COVID-19 exposure can be measured on two levels: direct exposure to the virus (i.e., the individual or a family member had COVID-19 or was in quarantine due to exposure to the virus), and indirect exposure (i.e., media exposure). For example, individuals who were directly exposed to the COVID-19 virus (e.g., either when individuals were in contact with people who then contracted with COVID-19 or were directly exposed to an infected individual) experienced greater mental distress [40]. A study that assessed the psychological impact of the COVID-19 lockdowns in Italy found that having a family member who was in quarantine was related to higher levels of anxiety and stress [41]. A recent review highlighted that being in quarantine was one of the most reliable predictors of symptoms of mental distress [42].

In terms of indirect exposure, there is robust evidence for an association between media exposure and various negative psychological outcomes during public crises [43]. For instance, the amount of time U.S. adults spent watching television on the day of the September 11^th^ attacks and the following days was correlated with symptoms of post-traumatic stress disorder [44]. Likewise, a survey conducted in China reported an association between exposure to COVID-19 information on social media and the prevalence of depression and anxiety [45]. In the U.S., increased exposure to a wider variety of media sources and spending more time on social media were linked to heightened levels of mental distress [46]. Another environmental factor that was likely to influence mothers’ psychological distress during the pandemic was the unpredictable opening and closing of schools [37]. During lockdowns, educational institutions often shut down, thus disrupting students’ routines and curtailing their support networks. Mothers were often forced to step in as teachers while working themselves [2]. Although some parents perceived the quarantine as a positive experience, mainly because it led to a closer relationship with their children [47], most mothers reported a considerable burden during the pandemic [2, 6]. Mothers who home-schooled their children during the COVID-19 outbreak reported higher levels of psychological distress than those who did not home-school or had no school-aged children [48].

### The current study

This study drew on Ecological Systems Theory [12] to examine the socio‐ecological determinants of maternal distress during the first year of the COVID-19 pandemic in Israel. The data consisted of individual‐level factors, family‐level factors, and environmental-level factors. Three general hypotheses were formulated, related to each socio-ecological level:

1. In terms of individual-level factors, younger mothers, as well as mothers who tend to use suppression more frequently and reappraisal to a lesser extent were predicted to report greater levels of psychological distress.
2. In terms of family-level factors, single mothers, mothers with more children in the family, mothers facing greater financial difficulties, having younger children or a child with DD were predicted to report greater psychological distress.
3. In terms of environmental-level factors, mothers whose children’s school was closed, mothers who were more closely exposed to COVID-19, or mothers who consumed more media related to the COVID-19 pandemic were expected to report higher mental distress.

## Method

### Procedure

The current study investigated maternal distress during the first year of the COVID-19 outbreak in Israel. Using a repeated cross-sectional design, data collection took place during two recruitment periods corresponding to peak periods of the COVID-19 pandemic in Israel. The first survey was administered from October 16 to November 15, 2020 (second lockdown). The second survey was conducted from January 16 to February 1, 2021 (third lockdown). The questionnaires were administered during two separate lockdown periods to explore the potential variations between them. This approach not only aimed to identify differences but also sought to increase the sample size, thereby facilitating a broader generalization of the results. During the periods when the questionnaires were administered, social distancing regulations, as well as restrictions on social gatherings, were in force. Almost all educational institutions were closed with the exception of certain special education schools, community educational institutions (for example in kibbutzim), and classes for children whose parents are essential workers (for example, schools in hospitals for children of medical staff). The participants completed an online survey administered via the Qualtrics platform (Qualtrics, Provo, UT). Before completing the questionnaire, they received a detailed explanation about the study and signed an informed consent form. The surveys were distributed via Facebook and school administrators. Participants who completed the survey were entered into a lottery to win a tablet, and two participants received a tablet at the end of data collection. The study was reviewed and approved by the University of Haifa IRB committee, license number: 286/21. All participants participated in the research voluntarily and anonymously and provided their informed consent to participate in this study.

### Participants

A repeated cross-sectional design was implemented, where each survey included a different sample of participants with similar socio-demographic characteristics. The inclusion criteria included being a mother above the age of 18, with at least one child younger than 18 living at home. An a-priori power analysis using G*power software indicated that a sample size of 123 participants was needed to detect a moderate effect size in a regression analysis with 80% power and an alpha of 0.05. Six hundred and twenty-five participants completed the questionnaire. The participants were recruited using a convenience sampling method. The final sample consisted of 575 mothers (Mean age 39.3, SD = 5.8).

### Measures

#### Individual-level factors

The individual-level factors were age and ER tendencies. Age was measured on a single open question where respondents were asked to indicate how old they were in years. Age was entered into the analysis as a continuous variable. Trait ER tendencies were measured using the Emotion Regulation Questionnaire (ERQ; [17]. The ERQ is made up of 10 statements that assess cognitive reappraisal (e.g., “I control my emotions by changing the way I think about the situation I’m in”), and expressive suppression (e.g., “I keep my emotions to myself”). Participants respond on a 7-point scale ranging from 1 (strongly disagree) to 7 (strongly agree). The Cronbach alpha internal consistency in the current sample was .85 for the reappraisal scale and .78 for the suppression scale.

#### Family-level factors

These factors included relationship status (in a relationship/not in a relationship), number of children in the family, household income (below/above average), child’s age (in years), and parenting a child with a disability (yes/no). Parents with multiple children were instructed to respond to the questionnaire with reference to a specific child of their choice. This approach was implemented to ensure that the responses were focused on the experiences and characteristics of a single child within the family, thereby maintaining the clarity and specificity of the data collected, as done in previous studies (e.g., Spinelli et al. [2]). Relationship status, household income, and the child’s disability were entered as categorical variables, while child’s age and the number of children in the family were entered into the analysis as continuous variables.

#### Environmental‐level factors

The environmental factors included school status, media consumption, as well as the amount of direct exposure to COVID-19. School status was composed of one question where parents were asked to report whether their child’s school had been closed or open the previous week. Media exposure was assessed by asking the number of hours daily that the respondents spent consuming media coverage of COVID-19. Possible exposure to COVID-19 was a categorical factor and was assessed using a checklist created for this study. The checklist was composed of five yes or no questions (e.g., "Have you been diagnosed with COVID-19?"; "Have you been tested for COVID-19?"; "Have you been in quarantine in the past month?", “Has someone close to you been diagnosed with COVID-19?”, “Has someone close to you died from COVID-19?”). A “yes” response to any of the items was coded as possible COVID-19 exposure.

#### Outcome variable

Psychological distress was evaluated on the Depression Anxiety Stress Scale (DASS; [49]. The DASS is a 21-item self-report questionnaire that assesses emotional distress by examining how often during the previous week the respondent experienced symptoms of depression (e.g., “I felt that life was meaningless”), anxiety (e.g., “I felt scared without any good reason”) and stress (e.g., “I found it hard to wind down”). The participants respond on a 4-point scale ranging from 0 (did not apply to me at all) to 3 (applied to me very much or most of the time). The Cronbach alpha internal consistency for the current sample was .90 for the depression scale, .86 for the anxiety scale, and .91 for the stress scale. Due to the high correlation between the different subscales, the total score was used in the analysis (Cronbach alpha for the entire scale = .95).

#### Data analysis

The data were analyzed using the Statistical Package of the Social Sciences (SPSS 25.0, IBM Corp.). Descriptive statistics and Pearson correlations were calculated for all variables. Then, a hierarchical regression technique was used to examine whether family-level factors and environmental factors accounted for a significant portion of the unique variance beyond that accounted for by individual-level factors. To assess the social-ecological perspective that underpinned this study [12], the ‘enter’ method within each block was used to determine the predictive strength of the individual-level factors (Block 1), family-level factors (Block 2), and environmental factors (Block 3) on parental distress. This made it possible to investigate the relative contributions of familial and environmental factors to mothers’ distress symptoms after the influence of individual-level factors had been considered. An alpha level of .05 was used for all statistical analyses. All data are publicly available via the Open Science Framework and can be accessed at https://osf.io/ka4v8/.

## Results

### Preliminary analyses

The participants reported having 2.5 children on average (SD = 1.0), with a mean age of 7.6 years (SD = 3.6); 21% of the mothers indicated having at least one child with a developmental disability. Most mothers had an above-average household income (77%) and 89% were married or in a relationship. Over half of the participants reported that their child’s school was closed at the time (second or third lockdown) they completed the survey (55%). Table 1 lists the descriptive statistics for all the study variables. The mothers in both lockdowns were similar on all variables except for direct exposure and media exposure to COVID-19 (*χ2* (1) = 23.41, *p* < .001, *χ2* (4) = 9.72, *p <* .05 respectively), indicating greater direct exposure during the third as compared to the second lockdown, but lower media exposure during the third vs. the second lockdown. School status also differed between lockdowns (during the third lockdown more schools were open; χ2 (1) = 9.89, *p* < .01).

**Table 1:** Descriptive statistics.

	Second lockdown	Third lockdown	Total	Statistics
	n = 310	n = 265	N = 575	
**Individual-level factors**				
Age [M (SD)]	39.6 (5.8)	39.0 (5.8)	39.3 (5.8)	t (573) = 1.09
Reappraisal [M (SD)]	4.6 (1.3)	4.5 (1.3)	4.6 (1.3)	t (573) = 1.43
Suppression [M (SD)]	2.9 (1.4)	2.9 (1.3)	2.9 (1.4)	t (573) = -0.13
**Family-level factors**				
relationship status [n (%)]				χ2 (1) = 2.26
*In a relationship*	271 (87%)	242 (91%)	513 (89%)	
Number of children [M (SD)]	2.5 (1.0)	2.5 (.9)	2.5 (1.0)	t (573) = -0.29
Household income [n (%)]				χ2 (1) = 1.79
*Below average*	66 (21%)	69 (26%)	135 (23%)	
*Above average*	244 (78%)	196 (74%)	440 (77%)	
Child’s age [M (SD)]	7.8 (3.6)	7.4 (3.7)	7.6 (3.6)	t (573) = 1.42
Child’s disability [n (%)]				χ2 (1) = 0.04
*Child has TD*	245 (79%)	210 (79%)	455 (79%)	
**Environmental-level factors**				
School status [n (%)]				**χ2 (1) = 9.89** **
*Closed*	188 (61%)	126 (48%)	314 (55%)	
*Open*	122 (39%)	139 (53%)	261 (45%)	
Direct exposure [n (%)]				**χ2 (1) = 23.41** ***
*possible COVID-19 exposure*	148 (48%)	179 (68%)	327 (57%)	
Media consumption [n (%)]				**χ2 (4) = 9.72** *
*Once or twice a day*	197 (64%)	152 (57%)	349 (61%)	
*2–5 times a day*	74 (24%)	57 (22%)	131 (23%)	
*6–10 times a day*	20 (6%)	37 (14%)	57 (10%)	
*10–20 times a day*	11 (4%)	10 (4%)	21 (4%)	
*More than 20 times a day*	8 (3%)	9 (3%)	17 (3%)	
**Outcome measure**				
DASS total score [M (SD)]	15.0 (11.3)	16.2 (10.6)	15.6 (11.0)	t (573) = -1.38

Note: *Bold results are statistically significant*

*p < .05.

**p < .01.

***p < .001. TD = typical development

Table 2 lists the bivariate correlations between all the study variables and measures of psychological distress. All the individual-level factors as well as household income, child age, media exposure, and school status were significantly correlated with psychological distress. The magnitude of the relationships between the predictor variables and psychological distress ranged from low (−.12) for both household income and school status to moderate (.25) for suppression.

**Table 2:** Pearson correlations.

	1	2	3	4	5	6	7	8	9	10	11
1. Reappraisal											
2. Suppression	.07										
3. Age	.04	.02									
4. Relationship status^a^	**-.11** **	-.03	**-.31** ***								
5. Number of children	.04	-.01	.03	**.21** ***							
6. Household income^b^	.02	-.05	.08	**.11** **	.03						
7. Child’s Age	.06	.08	**.57** ***	**-.19** ***	**.22** ***	.03					
8. Child’s disability^c^	.03	**.17** ***	**.12** **	**-.08** *	**.11** *	**-.11** **	.03				
9. COVID-19 Exposure^d^	-.01	-.03	-.10*	-.01	.01	-.04	-.06	.06			
10. Media exposure	-.04	.06	-.05	.01	.04	-.02	.06	**-.08** *	.07		
11. School status^e^	.06	.05	.01	-.01	.01	-.01	-.11*	**.40** ***	.01	-.10*	
12. psychological distress	**-.16** ***	**.25** ***	**-.13** **	-.00	**-.13** **	**-.12** **	-.08	.02	.03	**.16** ***	**-.12** **

Note: Bold results are statistically significant

a: 0 = single; 1 = in a relationship; b: 0 = below average; 1 = above average; c: 0 = child has typical development; 1 = child has a disability; d: 0 = no direct exposure; 1 = direct exposure; e: 0 = school closed;1 = school open.

*p < .05

**p < .01

***p < .001

### Predictive analysis

A hierarchical linear regression analysis was conducted to identify the significant predictors of maternal distress during the two COVID-19 lockdowns. The total DASS score served as the dependent variable. The first step, which included individual-level factors (age and ER strategies), accounted for 11% of the variance in maternal distress, *F*(3, 571) = 23.70, *p* < .001. In this step, all the variables significantly predicted maternal distress (see Table 3). The second step, in which family-level factors were added (including relationship status, household income, number of children, child age, and child disability), was significant, *F*(8, 566) = 10.76, *p* < .001, and accounted for 13% of the variance in maternal distress. This step added significantly to the model, accounting for an additional 2% of the variance in the maternal distress score, *F*change (5, 566) = 2.74, *p* > .05. In this step, the number of children in the family (*β* = .11, *t* = 2.56, *p* < .05) and household income (*β* = -.09, *t* = -2.13, *p* < .05) significantly predicted maternal distress. The third step, which included environmental factors (direct exposure to COVID-19, media exposure, and school status), was also significant, *F*(11, 563) = 10.17, *p* < .001, and accounted for a total of 16% of the variance in mothers’ psychological distress symptoms. This step added significantly to the model, accounting for an additional 3% of the variance in parental distress score, *F*change (3, 563) = 7.60, *p* < .001. In this step, media exposure (*β* = .13, *t* = 3.28, *p* < .001), and school status (*β* = -.13, *t* = -3.05, *p* < .01) significantly predicted maternal distress.

**Table 3:** Predictors of maternal distress: Hierarchical regression.

	Predictors	*B*	*S*.*E*	*β*	*t*	*p*	95% CI		VIF
							*LL*	*UL*	
***Step 1***							19.32	32.64	
	Reappraisal	-1.52	.34	-.18	-4.50	**< .001**	-2.18	-.85	1.01
	Suppression	2.10	.32	.26	6.54	**< .001**	1.47	2.73	1.01
	Age	-.25	.08	-.13	-3.28	**< .001**	-.39	-.10	1.00
***Step 2***							22.92	40.00	
	Reappraisal	-1.49	.34	-.18	-4.41	**< .001**	-2.15	-.83	1.02
	Suppression	2.06	.33	.25	6.32	**< .001**	1.41	2.69	1.05
	Age	-.25	.10	-.13	-2.66	**.008**	-.44	-.07	1.64
	Relationship status	-.77	1.53	-.02	-.50	.62	-3.77	2.23	1.22
	Number of children	1.23	.48	.11	2.56	**.01**	2.17	.29	1.16
	Household income	-2.21	1.04	-.09	-2.13	**.03**	-4.24	-.17	1.04
	Child’s Age	.04	.15	.01	.23	.82	-.26	.33	1.62
	Child’s disability	-.10	1.11	-.00	-.09	.92	-2.28	2.08	1.09
***Step 3***							20.37	37.83	
	Reappraisal	-1.38	.33	-.16	-4.14	**< .001**	-2.03	-.72	1.03
	Suppression	1.98	.32	.25	6.17	**< .001**	1.35	2.61	1.06
	Age	-.22	.09	-.11	-2.30	**.02**	-.40	-.03	1.66
	Relationship status	-.94	1.51	-.03	-.62	.53	-3.90	2.02	1.22
	Number of children	-1.27	.47	-.11	-2.69	**.007**	-2.20	-.34	1.16
	SES	-1.99	1.02	-.08	-1.95	**.05**	-3.99	-.01	1.05
	Child’s Age	-.07	.15	-.02	-.47	.64	-.37	.23	1.66
	Child’s disability	1.61	1.19	.06	1.35	.18	-.73	3.95	1.30
	COVID-19 Exposure	.25	.87	.01	.29	.77	-1.45	1.95	1.03
	Media exposure	1.42	.43	.13	3.28	**< .001**	.57	2.27	1.04
	School status	-2.87	.94	-.13	-3.05	**.002**	-4.72	-1.02	1.22

Note: Bold results are statistically significant; CI = confidence interval; LL = lower limit; UL = upper limit; VIF = variance inflation factor.

## Discussion

The COVID-19 pandemic is acknowledged to have constituted a severe psychological threat to individuals worldwide [1]. This study took a social-ecological approach to examine individual, familial, and environmental factors contributing to maternal distress during times of enduring stress. The findings showed that in terms of individual differences, the risk factors for psychological distress included being younger, as well as a greater tendency to use suppression and a lower tendency to use reappraisal. In terms of family-level factors, more children and lower household income predicted higher maternal distress. In terms of environmental factors, higher media exposure and school closure predicted higher psychological distress. The third regression model, which included all factors, revealed that all the variables remained significant predictors of psychological distress. Overall, this pattern of results underscores the importance of taking a wide range of factors into account when aiming to understand mothers’ distress during a crisis, including personal, familial, and environmental variables.

The results supported the first hypothesis by showing that being young, having a higher tendency to use suppression, and a lower tendency to use reappraisal predicted greater psychological distress in mothers. While it is possible that the elevated psychological distress of younger mothers could be explained by their first-time motherhood, there was no correlation between the number of children and the mother’s age, suggesting that first-time motherhood was less likely to account for this finding. Alternatively, the increased distress in younger mothers might be due to their inexperience with parenthood, whereas older mothers, who are more experienced, may have developed effective coping strategies for dealing with these challenges [26, 50]. Future intervention programs should consider providing training and assistance to young mothers on ways they can regulate negative emotions and cope with distress.

In terms of ER, participants who reported more frequent use of reappraisal also reported lower distress levels, whereas mothers reporting more frequent use of suppression reported higher psychological distress. These variables had the largest effect size. These findings are consistent with previous research showing that individuals who suppress their emotions tend to have more symptoms of anxiety, depression, and stress [17, 21]. By contrast, reappraisal might have helped mothers see the pandemic restrictions in a more positive light, find ways to use this time more productively, and therefore experience fewer symptoms of distress. Previous works have shown the beneficial outcomes of reappraisal during the COVID-19 pandemic [16, 18, 20, 24].

The second hypothesis concerning the influence of family-level factors on maternal distress was partially supported. Two family-level factors significantly predicted psychological distress: household income and the number of children in the family. Mothers reporting a lower than average income or those with more children had a greater risk of experiencing mental distress during economic crises such as the COVID-19 pandemic [27, 31]. A previous study found that during the pandemic, low-income parents found it difficult to afford extra childcare services and were unable to provide enough computers for their children’s online learning [27, 34]. Mothers had to help children with homeschooling, which may have also contributed to distress, especially in families with more children [4, 51]. These findings emphasize the unmet need to provide financial assistance to lower income families to reduce the enormous burden of COVID-19-induced psychological distress. Unlike other studies (for reviews see; [29, 30]), in this study, parenting a child with a disability, relationship status, and age of the child did not significantly predict mental distress in mothers. Parenting a disabled child was not associated with higher maternal distress, presumably because at the times the survey was conducted most special education schools were still open, whereas almost all regular education schools were closed. The reopening of special education institutions may explain the similar levels of distress in both lockdowns.

Interestingly, direct exposure to the COVID-19 virus did not predict maternal distress, despite studies suggesting that having a family member who contracted COVID-19 or being in quarantine were related to high anxiety and stress [41, 42]. An effect for this variable may not have been observed because the survey was during the second and third lockdowns, not the first, so that participants may have gotten used to the presence of COVID-19, and hence experienced less distress. Moreover, when distributing the questionnaires, individuals were presented with the chance to receive COVID-19 vaccination. This proactive measure could potentially have mitigated the psychological distress that relates to COVID-19 exposure [52]. However, it is noteworthy that studies conducted in Germany and France indicated that the second lockdown was correlated with higher levels of psychological distress compared to the first [52, 53].

The findings also showed that media exposure was a major predictor of maternal distress. Although individuals often turn to the media for information during a crisis to alleviate anxiety stemming from uncertainty [54], evidence suggests that repeated media exposure is more likely to increase anxiety through secondary traumatization [55] where exposure to the trauma of others can lead to anxiety and fear. In light of this finding, media companies should take ethics and humanistic considerations into account when covering a crisis event, especially when the public is already distressed. In addition, policymakers, public health campaigns, and other stakeholders bear the responsibility of ensuring that media information is accurate and not manipulated to induce fear or to gain more views.

School status (open or closed) was one of the strongest predictors of maternal distress during the COVID-19 period. Studies have shown that parents’ stress levels during school closures were significantly higher than before school closures [56]. Parents needed to oversee their children’s care and education at home for an indeterminate time, while having to work as well. School closure may have led parents to feel incompetent, stressed, and anxious about their child’s future [2, 35]. Hence, the closure of educational institutions should only be considered as a final recourse in crises. If such closures become inevitable, parents should be provided with the tools and support to facilitate their children’s education. This may involve offering online training for parents and requiring employers to adjust parents’ work hours when working from home.

### Strengths and limitations

The present study has a number of limitations. First, we relied on repeated cross-sectional data, which did not allow us to examine within-person changes in mental health. Therefore, we cannot draw causal inferences from the data. Second, the dissemination of the online survey via social networks may have limited the scope of the survey to specific population groups. However, there is growing evidence that supports the usefulness of social media platforms, especially in confined or difficult-to-access populations, such as parents of children with DD [57]. Addressing participants online helped us to obtain a large and varied participant pool, thereby boosting the study’s potential to draw valid conclusions and generalize the findings to a larger population. Third, children’s developmental disability was reported by the parents, and they were not required to provide or present a medical diagnosis. Furthermore, most special education institutions were open during the survey, while state educational institutions were mostly closed, which may be why having a child with DD did not predict maternal distress. Fourth, while this study extensively examined a diverse range of variables at the individual, familial, and environmental levels, thus providing a comprehensive understanding of the complex interactions between individual traits, family dynamics, and broader societal factors, the mothers’ work status was not examined. Given that mothers who had to work remotely during the COVID-19 pandemic were reported exhibited a decline in well-being [58], future research should incorporate this variable.

## Conclusion

This study applied Socio-Ecological Theory [12] to extend previous research on mental health risks and protective factors impacting maternal distress during the COVID-19 outbreak. We found that a variety of individual, familial and environmental factors could account for a significant amount of variance in mothers’ psychological distress during the pandemic. In particular, reappraisal emerged as an intra-personal resilience factor, while suppression emerged as a risk factor. These findings emphasize the importance of internal psychological resources in the ability to deal with crises. On the other hand, differences in levels of distress were also found in characteristics that are more difficult or cannot be influenced, such as age, socioeconomic status, and the number of children in the family. A better understanding of how socio-ecological factors affect mental health during the COVID-19 pandemic is critical to inform public policies aimed at reducing mental distress in a large and significant population such as mothers. For example, it would be beneficial to offer ER interventions, especially for parents who are at risk of distress, such as parents from low socio-economic backgrounds, or with a larger number of children. This strategic application of interventions aligns with the imperative to address both internal resilience factors and external socio-ecological determinants in promoting mental well-being. Additionally, considering the profound impact of the media on the mental health of individuals in crises, it is imperative for policymakers to utilize this influence responsibly. Rather than exacerbating anxiety, they should leverage the media as a force for good, by providing tools to help people cope effectively. Introducing stress management techniques, mental health centers, and economic support can serve as invaluable resources for empowering individuals to navigate crises with resilience and well-being. Finally, educational institutions play a significant role in mothers’ mental well-being. Thus, closing schools should be the last resort, and other less restrictive social distancing measures, such as wearing masks or studying in smaller groups, should be considered in the future. The immanent risks of climate change and population growth suggest there are likely to be other such crises in the future [59]. Therefore, finding the factors that increase resilience constitutes a necessary step to support humans’ physical and mental health. The findings here point to the importance of routine and educational frameworks, as well as the role of individual characteristics in times of crisis.
